# Forming three-dimensional closed shapes from two-dimensional soft ribbons by controlled buckling

**DOI:** 10.1098/rsos.171962

**Published:** 2018-02-28

**Authors:** Michio Aoki, Jia-Yang Juang

**Affiliations:** Department of Mechanical Engineering, National Taiwan University, Taipei 10617, Taiwan, Republic of China

**Keywords:** controlled buckling, shape transformation, kirigami, kamifusen, origami

## Abstract

Conventional manufacturing techniques—moulding, machining and casting—exist to produce three-dimensional (3D) shapes. However, these industrial processes are typically geared for mass production and are not directly applicable to residential settings, where inexpensive and versatile tools are desirable. Moreover, those techniques are, in general, not adequate to process soft elastic materials. Here, we introduce a new concept of forming 3D closed hollow shapes from two-dimensional (2D) elastic ribbons by controlled buckling. We numerically and experimentally characterize how the profile and thickness of the ribbon determine its buckled shape. We find a 2D master profile with which various elliptical 3D shapes can be formed. More complex natural and artificial hollow shapes, such as strawberry, hourglass and wheel, can also be achieved via strategic design and pattern engraving on the ribbons. The nonlinear response of the post-buckling regime is rationalized through finite-element analysis, which shows good quantitative agreement with experiments. This robust fabrication should complement conventional techniques and provide a rich arena for future studies on the mechanics and new applications of elastic hollow structures.

## 1. Introduction

Thin-walled structures are ubiquitous in nature, such as bamboo stems, decayed tree trunks [1] and arthropod exoskeletons [2], as well as in artificial structures, ranging from aircraft and storage vessels to artistic objects and toys. This is due to their unique merits of high strength-to-weight ratio, save of materials and weight economy. As such, several manufacturing processes have been developed to fabricate thin-walled structures across diverse areas of application, including injection [3], rotational [4] and blow moulding [5], as well as dip [6] and viscous coatings [7]. Although they have been widely used and proved indispensable in large-scale manufacturing, the requirement of complicated moulds and complex processes makes these techniques less applicable to settings where inexpensive, versatile and predictable rapid prototyping is desirable. Other limitations include the lack of theoretical models for predicting the wall thickness and uniformity of the structures due to the multi-physics complexity of the processes [7], and hence the optimization of process conditions often requires expensive and time-consuming trial and error. Viscous coating [7] was recently proposed to fabricate thin elastic shells by pouring a polymer solution on hemispherical moulds, which yields thin uniform shells whose thickness can be accurately predicted by a theoretical framework. However, it is unclear whether the same approach can be applied to fabricate structures other than hemispherical shells.

Here, we introduce a simple and robust method to form three-dimensional (3D) thin-walled structures with fully ‘closed’ surfaces, i.e. without openings or voids, by buckling two-dimensional (2D) elastic ribbons. An elastic ribbon is a slender elastic body whose cross section is flat and thickness is much smaller than its width [8]. Elastic ribbons exhibit interesting mechanical behaviour when subjected to a compressive, tensile or torsional load, and have been used to develop functional structures at various length scales [9,10]. Our present approach is inspired by the concepts of traditional Japanese art of *kirigami* and *kamifusen*. Kirigami is an ancient artistic technique, in which cut patterns are introduced into paper sheets to create a desirable and often complex 3D structure on folding [11–15]. Kamifusen is a traditional Japanese paper balloon, constructed from eight glassine pieces with the same shape, that can be turned into a 3D spherical shape by blowing air into it [16]. Kirigami has recently emerged as a viable tool for design and assembly of 3D mechanical structures from planar materials with special geometries and cut patterns [9,17–21]. Despite the extensive studies and a great variety of 3D structures reported using kirigami concepts, there is only limited study on how to create thin-walled structures with fully closed surfaces. Among those, Py *et al*. proposed an interesting manufacturing technique for fabricating closed shape surfaces based on elasto-capillarity, in which 3D structures were produced through the wrapping of a liquid droplet by a planar elastic sheet [22,23]. Here, we aim to demonstrate that fully closed 3D thin-walled structures of various shapes can be created by axially buckling a set of 2D elastic ribbons whose profile follows a particular design rule. Our approach offers a robust and predictable rapid-prototyping technique to form thin-walled structures of a wide variety of shapes and materials.

## 2. Results

### 2.1. Three-dimensional thin-walled structures with closed surfaces and the master profile

**Figure 1.**
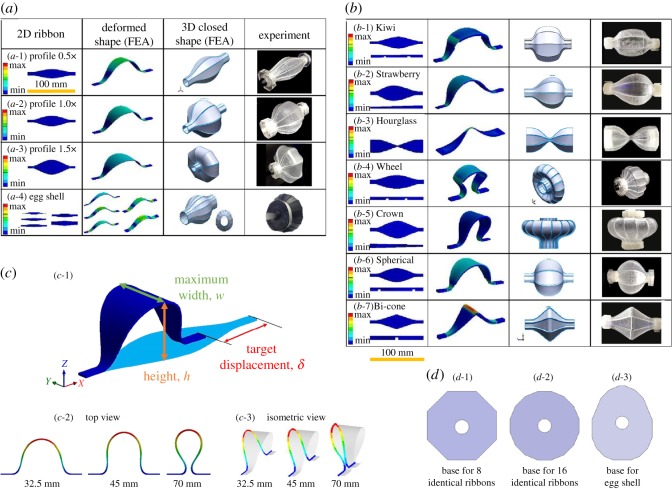
(*a*) Experimental and computational studies of 3D closed shapes using the master profiles, (*a*-1) Master profile 0.5× with eight identical ribbons, (*a*-2) Master profile 1.0× with eight identical ribbons, (*a*-3) Master profile 1.5× with eight identical ribbons, (*a*-4) the Eggshell profile, displacement 16.5 mm, eight ribbons with different widths. (*b*) Experimental and computational studies of various 3D closed shapes with ribbon profile modifications. (*b*-1) The Kiwi profile: two symmetrical line partial cuts, displacement 16.5 mm, eight identical ribbons, (*b*-2) the Strawberry profile: linearly varying thickness change from 2 to 1 mm, displacement 16.5 mm, eight identical ribbons, (*b*-3) the Hourglass profile: no specific pattern on the 2 mm surface, displacement 16.5 mm, eight identical ribbons, (*b*-4) the Wheel profile: two symmetrical line partial cuts, displacement 60 mm, 16 identical ribbons, (*b*-5) the Crown profile: linearly varying thickness from 2 to 1 mm, displacement 60 mm, 16 identical ribbons, (*b*-6) the Spherical profile: two symmetrical line partial cuts, displacement 16.5 mm, eight identical ribbons, (*b*-7) the Bi-cone profile: one line partial cut, displacement 16.5 mm, eight identical ribbons. (*c*) Schematic of the deformed ribbon: target displacement *δ*, height *h* and maximum width *w.* Buckled ribbons at three different displacements. (*d*) Three base designs for the assemblies consisting of eight, 16 identical ribbons and eight ribbons with different widths (the Egg profile). Colour code indicates the maximum principal strain (FEA results).

**Figure 2.**
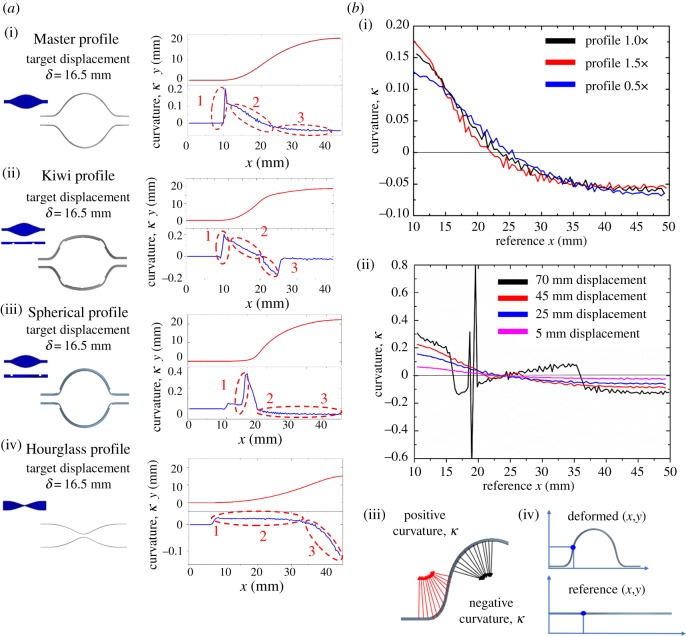
Curvature analysis. (*a*(i)) The Master profile, (*a*(ii)) the Kiwi profile, (*a*(iii)) the Spherical profile and (*a*(iv)) the Hourglass profile. (*b*(i)) Comparison of curvature among Master profile 1.0×, Master profile 0.5× and Master profile 1.5×. (*b*(ii)) Comparison of curvature at different displacements. (*b*(iii)) Definition of positive and negative curvatures *κ*. (*b*(iv)) Expression of reference coordinate (*x*,*y*) and deformed coordinate (*x*,*y*).

**Figure 3.**
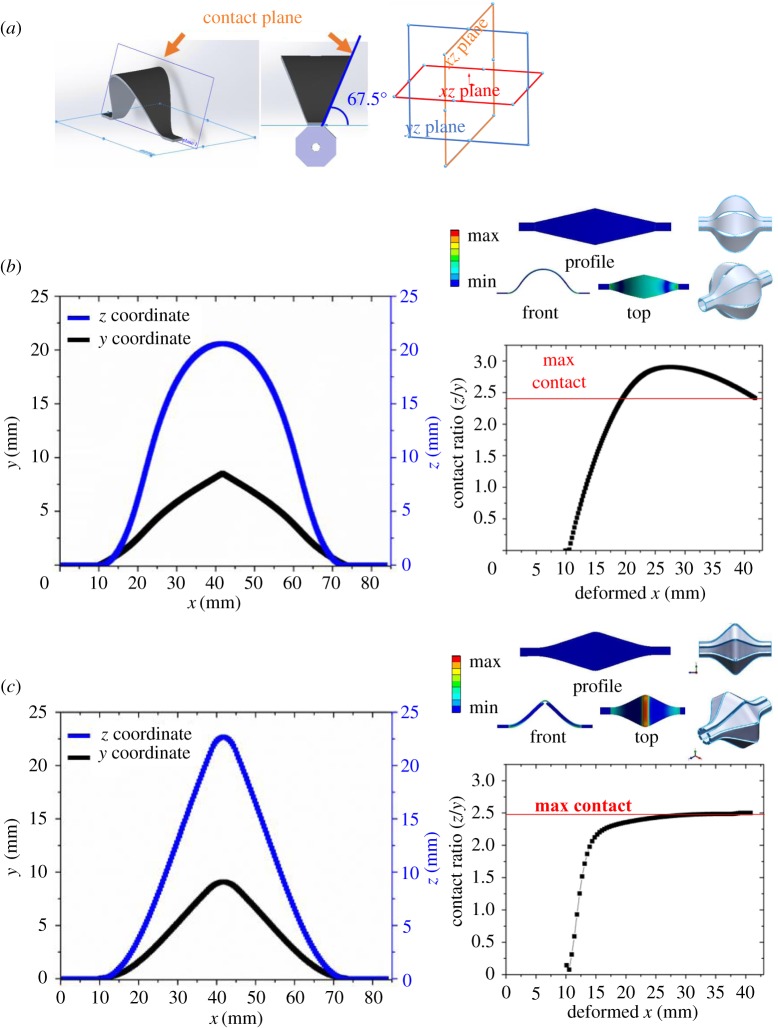
Assembly principles, contact plane and contact ratio, *CR *≡ *z*/*y*, where *y* and *z* are the deformed coordinates. (*a*) Schematic showing the top ribbon and the angle *θ*_c_ = 90°–360°/8/2 = 67.5°, corresponding to *CR *≈ 2.4. The edges of the deformed ribbon must fully lie on the contact plane for a perfect assembly. The other seven ribbons are not shown for clarity. (*b*) Assembly of eight identical ribbons using an arbitrary profile. The *CR* is not constant and the 3D structure shows gaps between adjacent ribbons. This is an example of poor assembly. (*c*) Assembly of eight identical ribbons using an optimized profile with a more uniform *CR*. This ribbon is derived from the one in (*b*) with an additional line partial cut in the *y* direction and profile fine-tuning. This is an example of good assembly.

**Figure 4.**
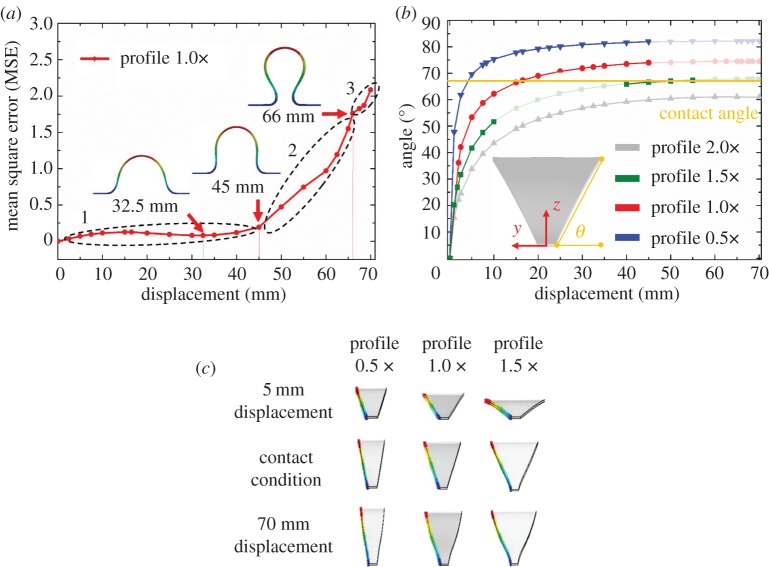
Assembly quality. (*a*) The mean squar error (MSE) of the distance between the actual deformed edge and the contact plane as a function of the displacemnt for Master profile 1.0×. (*b*) Relationship between the angle *θ* and displacement. *θ* must be equal to *θ*_c_ (= 67.5° for eight ribbons). The regions where MSE is larger than an acceptable threshold value are shown in transparency. The target displacements for different profiles are different and are determined at *θ *= *θ*_c_. The inset (profile 2.0×, displacement = 20 mm) shows the definiton of *θ*. (*c*) FEA simulations showing how straight the edge is at different displacements for different profiles.

Figure 1*a*,*b* presents various 3D thin-walled structures with closed surfaces, each transformed from corresponding eight or 16 pieces of 2D elastic ribbons of elastomer, such as polydimethylsiloxane (PDMS, transparent) and thermoplastic olefin (TPO, black colour). Those ribbons are fabricated by a standard casting process [24–26] (see Material and methods) with a variety of patterns engraved on the surfaces to enhance flexibility in certain orientations and/or specific locations [17]. The primary patterns used are line partial cuts and linearly varying thickness. Line partial cuts are shallow recessions, created on a ribbon surface, with properly designed location and recession depth. The width of the profiles is determined according to the desired 3D closed shapes. One end of the ribbon is attached to a fixed base; the other end is attached to a movable base, which can move along the *x* direction. We then apply a compressive force on the movable base to induce the ribbon to buckle out of the plane, forming the target 3D configuration (electronic supplementary material, figure S8). In this study, each 3D closed shape is composed of eight or 16 ribbons, but other numbers of ribbons may also be used. Also, the ribbons that are used to form a 3D shape may not be identical. For example, the ‘Egg’ profile consists of 16 ribbons of different widths as shown in figure 1*a* (*a*-4).

**Figure 5.**
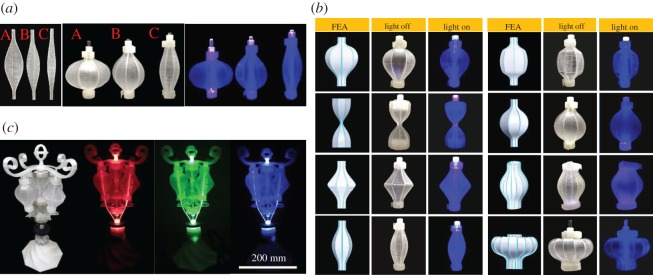
Soft light bulbs. (*a*) Comparison of three different ribbon profiles, and their corresponding light bulbs. An external LED light source is placed at the bottom of the light bulbs. Light passes through the deformed ribbons by total reflection. Light bulbs contain no internal light source. (*b*) Soft light bulbs of various shapes. FEA results are in good agreement with the experimental results. (*c*) LED lamp with soft light bulbs.

**Figure 6.**
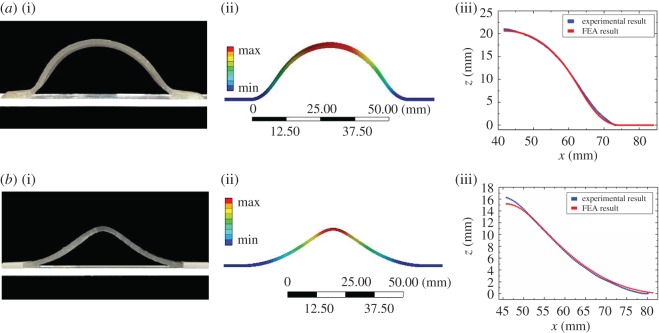
Comparison between FEA simulations and experiments. (*a*) The experimental image of the deformed Master profile (i); FEA result of the deformed Master profile (ii); comparison between the experimental and FEA results (iii). (*b*) Experimental image of the deformed Hourglass profile (i); FEA result of the deformed Hourglass profile (ii); comparison between the experimental and FEA results (iii).

The profiles of the ribbons in figure 1 are all derived from the ‘Master profile 1×’ as shown in figure 1*a* (*a*-2). We refer it as the Master profile 1×, or simply the Master profile, because the maximum lengths of the transformed 3D shape are equal in the *x* and *z* directions (figure 1*c*). The fixed base is included when calculating the length, so the actual length along the *z* direction is shorter than that in the *x* direction. Master profile 1× can be used to generate various 3D closed shapes by simply scaling up or down the profile width (in the *y* direction) in a range between 0.5× and 1.5× as shown in figure 1*a*. Therefore, it is a key ribbon profile to turn 2D precursors into 3D closed shapes. Figure 1*a* (*a*-1) and (*a*-3) illustrates the Master profile 0.5× and 1.5×, which are created by multiplying the width of the ribbon by 0.5 and 1.5, respectively. We define the longer diameter of the transformed shape as the major axis and the shorter one as the minor axis. For the Master profile 0.5×, the *x* axis is the major axis, and it requires a displacement of approximately 4.8 mm, referred to as the target displacement *δ* (figure 1*c*), to form a closed shape. On the other hand, the major axis of the Master profile 1.5× is the *z* axis with *δ *≈ 45 mm. Note that these master profiles can only be applied to create relatively simple 3D structures (figure 1*a*). A wider variety of 3D shapes, however, can be created based on the same master profile but with minor modifications in the form of engraved patterns on the ribbon surfaces and/or varying ribbon thickness. Figure 1*b* presents 2D precursors with engraved patterns or varying thickness, and their corresponding 3D shapes. Figure 1*b* (*b*-1), the ‘Kiwi’ profile, features two line partial cuts at 25 and 75 mm positions. The pattern yields an irregular bulge in the deformed ribbons as a result of sudden curvature changes near the partial cuts. The patterns not only influence local deformation but also determine the entire 3D shape. For example, both the Kiwi and the Spherical profiles (figure 1*b*, *b*-6) have two line partial cuts, yet their deformed 3D shapes are completely different, indicating that the location of a line partial cut is crucial to determine the final deformed shape.

We used the finite-element analysis (FEA) package ANSYS Workbench to simulate and elucidate the post-buckling behaviour of 2D ribbons with nonlinear large deformation effect included. We consider nonlinearity due to large deformation and assume linearly elastic material properties. Our numerical simulations and experimental results are in good quantitative agreement, indicating that the modelling approach and assumptions are adequate (see Material and methods). The shape transformation is performed by applying a force to the movable base towards the fixed base until the target displacement is reached (figure 1*c*). For the case of PDMS, we use an elastic modulus *E* *=* 2.46 MPa and a Poisson ratio *ν* *=* 0.41 according to standard tensile tests [27]. The four-node shell element (Shell 181) is used for cases with uniform thickness. For ribbons designed with engraved patterns or non-uniform thickness, we used quadratic hexahedral 3D solid element (Solid 186). Both element types support nonlinear effects [28,29]. A refined element size of 0.5 mm with quadrilateral mesh type is assigned to each ribbon; a further decrease in element size does not change the simulation results. The linear buckling analysis is first carried out to investigate the results under different buckling modes. Among those, the mode with the lowest critical load is the one we expect to observe experimentally and hence is used in the post-buckling simulation. In addition, the linear buckling analysis also reveals critical buckling points. Our FEA simulations exhibit excellent quantitative agreement with the e*x*perimental results, thereby establishing computation as a powerful means for rapid design iterations.

### 2.2. Curvature control by strategical patterning

The characteristics of the deformed shape can be analysed by examining the curvature *κ* of the ribbon along the *x* axis (figure 2). As shown in figure 2*b*(i), the Master profile 1.5× creates the largest positive curvature near the fixed base and the smallest negative curvature near the centre, whereas the Master profile 0.5× creates the smallest positive curvature near the fixed base and largest negative curvature near the centre. This indicates that the size of maximum width plays an important role in determining the deformed curvature patterns. Moreover, the reflection point of the Master profile 1.5× is located at 22.5 mm, which is closer to the fixed base than those of profiles 1.0× and 0.5×. As such, the position of reflection point can be specified by properly designing the profile width. We also observe that the reflection point remains at the same position for the same profile regardless of the amount of displacement, while the curvature increases with the displacement (figure 2*b*(ii)).

A careful investigation of the Master profile (figure 2*a*) reveals that its curvature can be divided into three different regions: (1) rapidly increasing, (2) moderately decreasing (at positive curvature) and (3) slowly decreasing (at negative curvature). We define that the curvature *κ* is positive when the curve turns downwards and is negative when turning upwards as illustrated in figure 2*b*(iii,iv). The rapid increase in region 1 is because of the horizontally fixed base, which results in a deformation in the *z* axis and a larger curvature occurred near the fixed base. In region 2, the curvature continues to decrease and the positive curvature starts turning upwards. The reflection point is located at *x* = 23 mm. In region 3, the slope gradually decreases towards the central part of the ribbon. We then consider the patterns needed for a ribbon to achieve the target curvature design. For instance, the decreasing rate is milder in region 3, and if we aim to make a rapid decrease in this section, what kind of modification is effective? To answer this question, we provide the pattern called ‘Kiwi’, inspired by the shape of the fruit (figure 2*a*). This profile contains the pattern with a line partial cut at *x* = 25 mm and has a reflection point at the centre. The partial cut triggers abrupt curvature decrease in region 3, and turns positive curvature into negative at a smaller *x* compared with the Master profile. As another example, if we aim to obtain a spherical shape, i.e. the deformed shape has a uniform curvature, what modification is required? The Spherical profile is obtained by placing a line partial cut at a position very close to the fixed base. Compared to the Master profile, the curvatures near the fixed base surge rapidly because larger deflections are generated in these sections. Hence, the position of partial cuts dramatically changes the deformed shape. Our next attempt is to make a curvature pattern completely opposite to the master profile. The profile, ‘Hourglass’, has a narrow centre on the ribbon. The wider width creates a relatively small curvature; hence, if we aim to make a smaller curvature at region 1 and a large curvature at region 3, the Hourglass profile is effective.

Asymmetrical shapes, such as ‘Strawberry’ and ‘Crown’ in figure 1*b*, may also be created by a similar approach. Both shapes use 2D ribbons with linearly varying thickness from 2 to 1 mm. Another profile called ‘Bi-cone’ presents zero curvature at almost every position except for the central part (figure 1*b*, *b*-7). This profile has a line partial cut at the centre of the ribbon, designed to induce a substantial local deformation near the centre while maintaining zero curvature elsewhere. We have demonstrated, accordingly, that modifications of the partial-cut location, size and side of a surface are essential to achieving our target curvature patterns.

### 2.3. Assembly principles and contact ratio

What are the conditions that an assembly of deformed ribbons must meet in order to form a fully closed surface? Consider the top ribbon of an assembly of eight identical ribbons (figure 3*a*). Its contact plane with the adjacent ribbon must form an angle of *θ*_c_ = 90°−360°/8/2 = 67.5° with respect to the horizontal *xy* plane. Note that *θ*_c_ is only dependent on the number of ribbons in an assembly. The coordinate of every contact positions of the deformed ribbons must be located on this plane in order to form a fully closed 3D structure. This can be achieved if both of the following conditions are satisfied: (i) the profile's *y* and *z* coordinates reach an angle of *θ* *=* *θ*_c_, where *y* and *z* are the coordinates of the ribbon's edge at the deformed configuration, and (ii) the contact ratio, defined as *CR* = *z*/*y* *=* tan(*θ*), is constant along the *x* axis

Figure 3*b* shows the top (*xy* plane) and front (*xz* plane) views of the deformed profile of an arbitrary ribbon and its corresponding *CR*. A closer examination of the assembled 3D structure reveals multiple gaps and overlaps between adjacent ribbons, indicating that such a 2D ribbon design cannot be transformed into a 3D structure with a fully closed surface. This is because its *CR* is not constant along the *x* axis. In this case, the two deformed adjacent ribbons touch at the centre (*x *≈ 42 mm) with *CR* = tan(*θ*_c_) = tan(67.5°) ≈ 2.4; the region where *CR* > 2.4 indicates an overlap, whereas the region where *CR* < 2.4 indicates a gap. However, a fully closed 3D surface can be created by applying minor modifications to the profile in figure 3*b*. The modified profile features a line partial cut at the centre of the profile along the *y* axis as well as fine-tuning of the width at specific locations (figure 3*c*). Now, the resultant 3D structure exhibits a closed surface, which is attributable to a relatively uniform *CR* along the *x* axis. The quality of assembly improves as the region of constant *CR* increases.

If the ribbon is engraved with partial cuts in both the *x* and *y* axes, the contact positions no longer stay on a plane because the ribbon also induces deformations along the *y* axis. In this case, the same assembly principles still apply, but it may involve more design iterations to obtain the corresponding fully closed surface.

### 2.4. Assembly quality as a function of displacement

We described the assembly principles for forming 3D closed surfaces in the previous section. Here, we present a procedure to assess the assembly quality of the assembled structure. We define a perfect assembly as the case where the interface of any two adjacent ribbons exhibits no gaps and overlaps. For cases where the assembly is not perfect, we quantify the assembly quality by evaluating mean square error (MSE) [30] of the distance between the actual deformed edge and the contact plane as illustrated in the inset of figure 4*b*. The MSE of a perfect assembly is zero.

Figure 4*a* shows the MSE as a function of the displacement for Master profile 1×. The curve may be divided into three regions: (1) 0–45 mm, the curve is relatively flat and small in magnitude. When the displacement is zero, the ribbon is undeformed and two adjacent ribbons are in perfect contact without any gaps or overlaps, i.e. MSE = 0. (2) 45–66 mm, the value rapidly increases with the displacement. At a displacement of 45 mm, the deformed edges become perpendicular to the bases (figure 4*a*), resulting in a rapid increase in MSE in this region. (3) 66–67.5 mm, the rate of increase is reduced. The deflection *h* reaches the maximum value and starts to decrease at a displacement of 66 mm. Similar trends are also observed in the profile 1.5× and the profile 0.5× (see electronic supplementary material for details).

Figure 4*b* shows the relationship between the displacement and angle *θ*, as defined in the inset. As described earlier, one condition to form 3D closed surfaces is that the angle *θ* must be equal to *θ*_c_. For the case of eight identical ribbons, the 3D closed surfaces occur at *θ* = *θ*_c_ = 67.5°, and the target displacements are 4.8, 16.5 and 45.3 mm for profile 0.5×, 1.0× and 1.5×, respectively (figure 4*b*). We highlight two key observations. First, the curve for profile 2.0× does not reach *θ* = 67.5°, and hence profile 2.0× cannot be used to form any 3D closed surface. Second, for the profiles 0.5×, 1.0× and 1.5×, some displacements may generate an MSE that is greater than a threshold value specified by the user. The edge of the deformed shape under such a displacement is not straight and cannot form a 3D closed surface (e.g. figure 4*c*, 70 mm displacement). Those regions that have an unacceptable MSE are shown in transparency in figure 4*b*.

Figure 4*c* compares the edges of the deformed ribbons, calculated by FEA, at different displacements. The result shows that the edges are straight at the respective target displacements, and are not straigtht at a large displacement of 70 mm, corresponding to regions 2 and 3 in figure 4*a*.

### 2.5. An example of new application: soft light bulb

We apply the established design concept and principle to create soft light bulbs. This unique forming method allows one to design and assemble a variety of soft light bulbs of different 3D shapes (figure 5*a*,*b*). Each soft light bulb is composed of eight transparent PDMS ribbons, and the shape transformation is achieved by buckling. Figure 5*c* shows a representative embodiment of a lamp, consisting of several soft light bulbs and an external light-emitting diode (LED) light source, located within the base. The frame and base of the lamp, made of transparent poly(lactic acid) and acrylic, are manufactured by 3D printing (electronic supplementary material). The light bulbs are illuminated based on the principle of total reflection. Light enters the light bulbs from their bottom end, passes through their curved surfaces via total reflection and exits at the top end.

As PDMS is highly elastic, the shape transformation is reversible and repeatable, and the transformed 3D thin-walled structure is not as fragile as the conventional light bulbs, offering maximum safety. This provides a new lighting concept for personal entertainment. The manufacturing cost is relatively low, and no expensive tools for machining and glass blowing are required. Current light bulb manufacturing process is fully automated in a factory. The glass bulbs are blown by a machine called a ‘ribbon machine’. Although suitable for large-scale industrial applications, this process cannot be done by individuals for rapid prototyping at residential settings; our present study provides an alternative to meet such needs.

## 3. Discussion

In this study, we utilize ribbons of elastomer of predefined shapes and compress them until they deform out of plane. By combining multiple of these ribbons (8 or 16 pieces), we can make a closed 3D thin-walled structure that resembles a hollow ellipsoid. In addition to controlling the 2D shape of the ribbon, there are some partial cuts or varying thickness that help guide the transformation to form a wide variety of 3D structures. Over the last two decades, the use of origami/kirigami principles to transform 2D materials into 3D shapes has inspired many studies and applications, pioneered by Rogers and co-workers [17,31–36] and other researchers [9,10,20,21,37–40]. The main difference between the present study and prior work is that we showed that a broad set of ‘closed’ 3D shapes could be created by a systematic approach, whereas the transformed 3D structures of almost all prior work have ‘open’ surfaces.

Although thin elastic shells made of soft elastomers, such as vinylpolysiloxane, can be fabricated by conventional moulding process [41], it requires a two-part 3D mould of the target shell structure, and the male and female parts of the mould must be perfectly aligned during casting. Also, the adhesion between the shell and both parts of the mould becomes very strong for some materials, such as PMDS; it is difficult to separate them without damage during the release step. By contrast, our method does not require a 3D mould. Instead, only a planar negative mould is required to fabricate the ribbons, with the PDMS poured into it and filling the regions left open by the mould [25] (electronic supplementary material, figure S17). The release of ribbon from a planar mould is much easier than the case for conventional 3D moulding, and hence the present method can be used for a wider range of elastomers with various surface and mechanical properties. Moreover, the fabrication of ribbons is not restricted to PDMS moulding. For example, the ribbons in electronic supplementary material, figure S8, were obtained from a TPO thin sheet by cutting.

Our FEA modelling shows remarkable agreement with the experimental results in predicting the transformed 3D shape from a given 2D shape (figure 6). However, the modelling is less predictive for the inverse design problem, i.e. it cannot predict what 2D profile is needed to form the 3D shape—this is an interesting topic for future research. Nevertheless, the family of Master profiles presented here is the basic 2D profiles for forming relatively simple ellipsoidal shapes (figure 1*a*). For more complicated shapes, one may refer to the samples listed in figure 1*b* as a starting point for design iteration.

Although the 2D ribbons (length = 100 mm, thickness ≈ 1 mm) used in this study are large compared with the cases in the review paper [31], our FEA modelling and findings should be applicable to smaller and larger sizes. Similar modelling has been applied to study the buckling of microscale origami structures and showed excellent agreements with experiments [31,34]. The assembly principles for the ribbons to form a closed surface remain the same irrespective of ribbon size. The thickness-to-length ratio will also affect the buckled shape. For a given profile and material, there is a lower limit for the ribbon thickness, below which the bending stiffness of the ribbon is too small to resist any bending moment.

## 4. Conclusion

Inspired by the traditional arts of kirigami and kamifusen, we demonstrate that 3D thin-walled structures with a variety of closed surfaces can be created by buckling planar thin elastic ribbons. Such a shape transformation is rapid, reversible and repeatable, and can be accurately predicted by FEA. We identify the design and assembly principles that must be satisfied in order to form a closed surface. We found a class of master profiles that meet those principles and are used to derive ribbons that generate more complex 3D structures. We extend this design concept and propose a new application—soft light bulbs. Unlike a conventional light bulb, the soft light bulb is made of transparent elastomer and is illuminated when the light of an external source passes through its surface by total reflection. The robustness and flexibility of this approach are inherent consequences governed by the elasticity in the nonlinear regime. The generality of this framework should provide a rich arena for future studies to fabricate 3D thin-walled structures in a variety of other geometries. Furthermore, our design concept may be important in the ongoing revival of the stability of thin elastic ribbons, in particular because it utilizes previously less explored design space in the post-buckling regime with strong geometric nonlinearities [8].

## 5. Material and methods

### 5.1. Fabrication of two-dimensional ribbons

Two different elastomer materials are used in our experiments: PDMS and TPO. PDMS is a material which exhibits great linear-elastic behaviour and can be extended for more than 20% in strain [27]. To compose PDMS, Sylgard 184A and Sylgard 184B are mixed in a form of liquid and poured into a mould [24]. The special mould is pre-designed and created using a 3D printer. The elastic properties of PDMS can be tuned by applying different weight ratios of Sylgard 184A and Sylgard 184B. A weight ratio of 10 : 1 (A : B) is used in this study. Another material adopted is TPO sheets, which are a black rubberlike material and are used to build designs requiring uniform thickness without patterns. On the other hand, PDMS ribbons with engraved patterns and non-uniform thickness can be fabricated by a simple moulding process.

### 5.2. Experimental procedure

The experimental procedure is illustrated in electronic supplementary material, figure S8. To demonstrate the assembly and buckling process, we designed a simple experimental device, which consists of two bases and one rod connected to the frame (figure 1*d*). The bottom base is fixed, and the top base is allowed to slide towards or away from the bottom base. A set of 2D ribbons are first pre-assembled on both bases in 3D space and are then pressed until they bend out of plane to form the desired thin-walled structure. The determination of the displacement, i.e. the pushing distance, required to form a closed surface is described in the main text.

### 5.3. Comparison between simulation and experimental results

As stated earlier, our simulations (ANSYS Workbench) and experimental results show good quantitative agreement. Figure 6 illustrates two representative cases: the Master profile and the Hourglass profile. The deformed coordinates of the simulation results are extracted by the curve fitting method using the commercial packages Matlab; the experimental data are retrieved from the high-resolution images.

### 5.4. Curvature analysis

Curvature *κ* is calculated at the centre of every two adjacent nodes by applying the general curvature formula:

$$\frac{1}{\rho }=\frac{{\text{d}}^{2}y\text{/d}x^{2}}{{\left[1+{\left(\text{d}y\text{/d}x\right)}^{2}\right]}^{3/2}\;}.$$

Firstly, we assign 400 nodal positions along the *x* axis with a mesh size of 0.25 mm. A total number of 398 centre spots are retrieved between every two nodal positions. We then calculate the curvatures at each centre spot. The curvature of the initial (*x* = 0 mm) and the final nodal position (*x* = 100 mm) cannot be calculated; however, both positions are fixed to the base so their curvature must be zero.

## Supplementary Material

Supplementary information and figures

## Data accessibility

The data generated or analysed during this study are included in this published article (and its electronic supplementary material).
