# Supplementary material for: Forming three-dimensional closed shapes from two-dimensional soft ribbons by controlled buckling
Source: R Soc Open Sci. 2018 Feb 28;5(2):171962. doi: 10.1098/rsos.171962 (PMC5830783; doi:10.1098/rsos.171962)
Supplement: Supplementary information and figures [file rsos171962supp1.pdf]

Supplementary Material for
Forming three-dimensional closed shapes from two-dimensional soft ribbons by controlled buckling

Michio Aoki and Jia-Yang Juang*

Department of Mechanical Engineering

National Taiwan University

Taipei 10617, Taiwan

*Corresponding author: jiayang@ntu.edu.tw

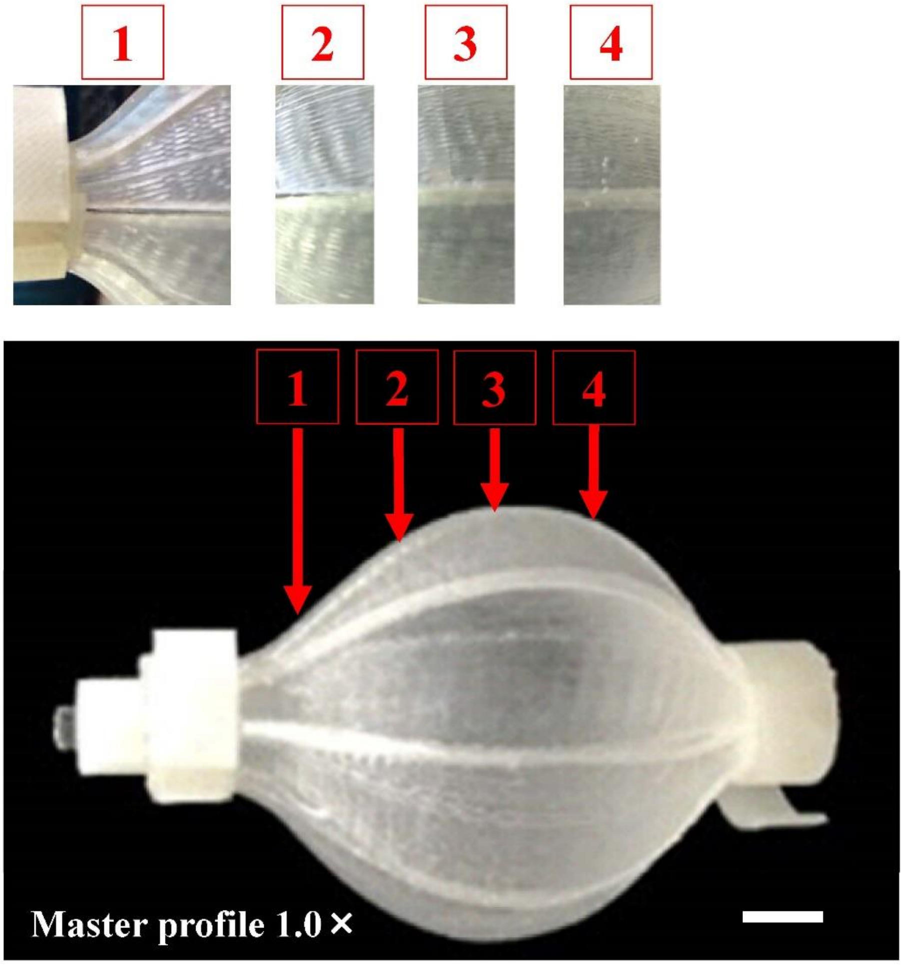

Figure S1. Images of the experimental structure zooming at the boundary between two adjacent ribbons at four locations. They all show good contact conditions with slight gap at location 1. The scale bar is 10 mm.

The Figure S2 shows the delflection in three different porfiles (Profiles 1.5×, 1.0× and 0.5× are illustrated). The result shows the larger profile (Profile 1.5×) reduced the size of maximum deflection. On the other hand, smaller profile slightly increased the maximum deflection compared with the master profile 1.0×. The difference of maximum deflection is relatively small in the case of small displacements. The deviation gradually increases as the displacement elevates. As a result, scaled down profiles have more chances to generate a closed shape based on the master profile since it only requires a small displacement. If the deformed configuration was very different form the master profile 1.0×, and the profile cannot form a closed shape. As a conclusion here, the scale down master profile (to 0.5× or even smaller), the deformed configuration will make smaller changes. The scale up the master profile (Range between 1.0× to 1.5×), the deviation will increase. However, we observed the deviation is within the acceptable range in the case of profile 1.5×. Hence, our master profile can be used in the range between 0.5× to 1.5×.

The maximum deflection increased rapidly in initial displacement stage. (Range between 0 – 20 mm) The increment of the maximum deflection slows down above 45 mm displacement due to the ribbon deform perpendicular to the ground. At 66.5 mm displacement, the maximum deflection reach to the peak and start reducing after passing this point. Also the difference of the deformed configuration is getting bigger, once the

displacement is bigger.

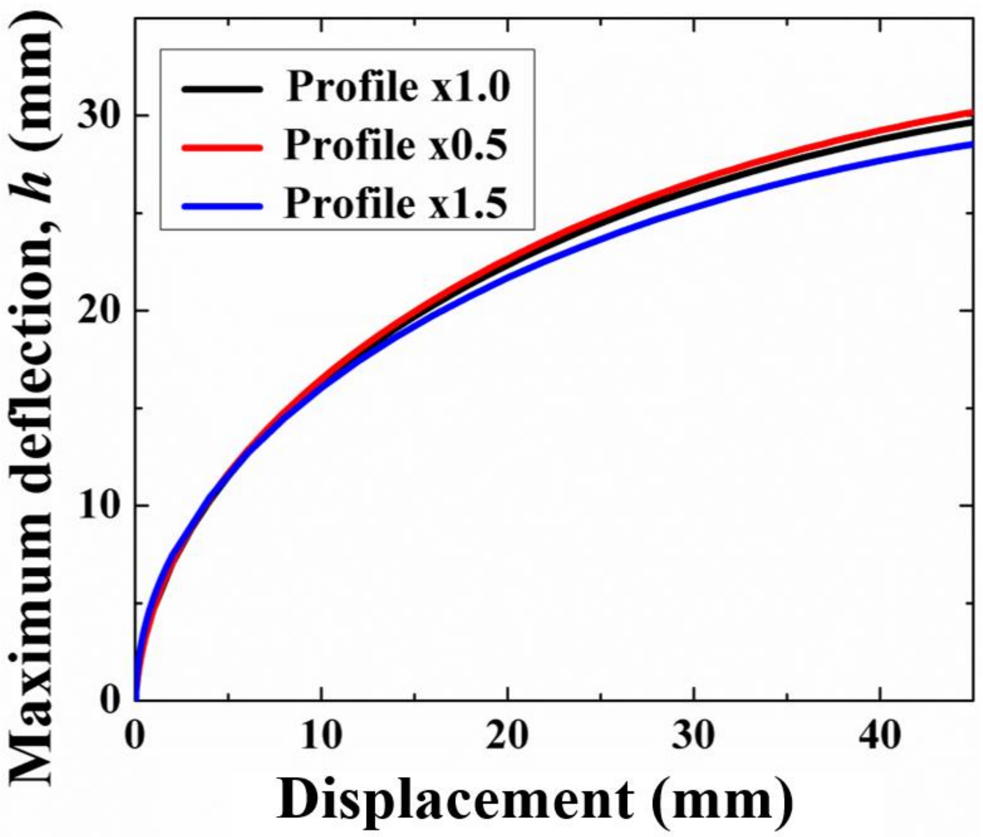

Figure S2. The maximum deflection in three different profiles. (Profile1.5×, 1.0× and 0.5× are illustrated) The result shows the scaled up profile minimize the maximum deflection.

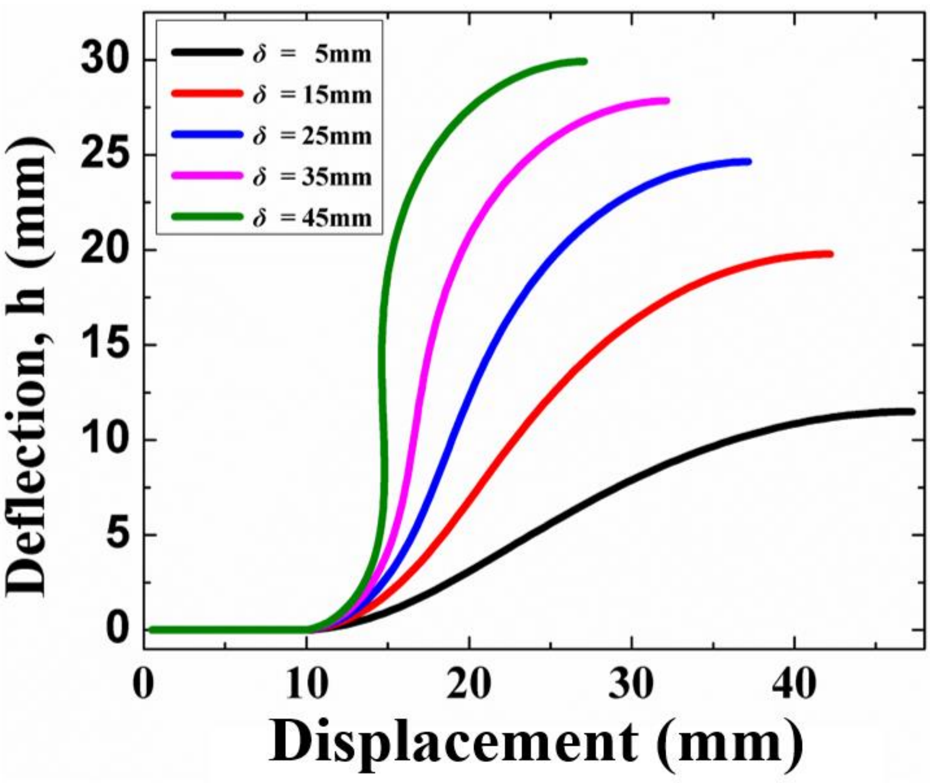

Figure S3. Deformed configuration in different displacements. The maximum deflection increased rapidly in initial stage. (Displacement between 0 – 20 mm) The increment of the maximum deflection is slows down above 45 mm displacement.

We analzed the target displacement of five different profiles to estimate the relationship between the target displacement and the maximum deflection. We knew the how much maximum deflection needed since we designed our 2D precursor. Therefore, if we know the target displacement, we can generate any size of profile within the range between 0.5× to 1.5×. (The curve fitting and equation are calculated using commercial packages from MATLAB).

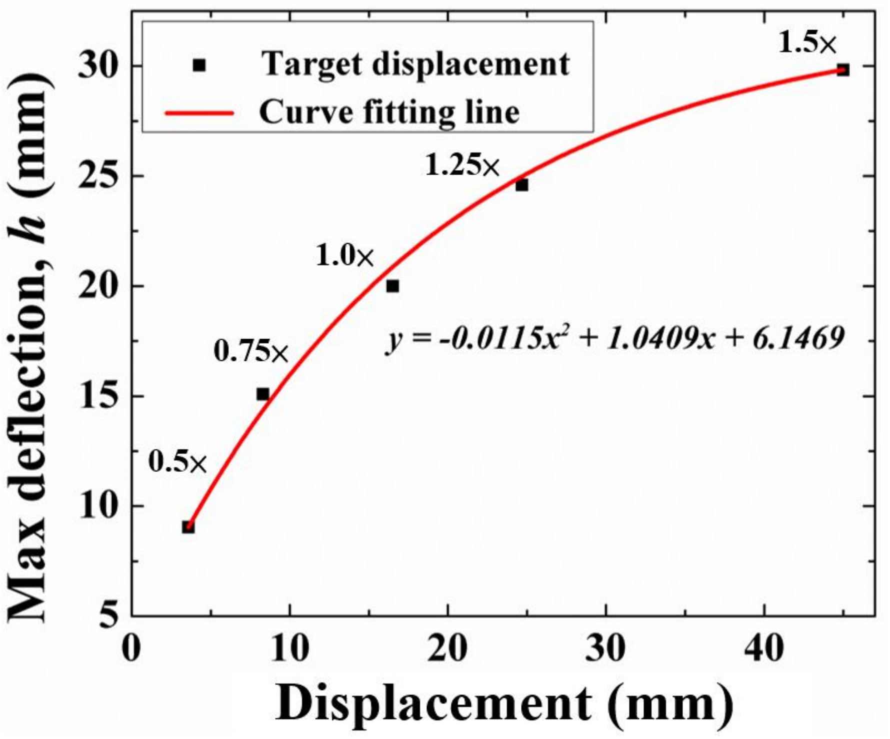

Figure S4. Relationship between the target displacement and the maximum deflection. The curve predicts the target displacement in range between profile 0.5× to 1.5×. The equation of $y = -0.0115x^2 + 1.0409x + 6.1469$ can be applied for observing the target displacement in different profiles.

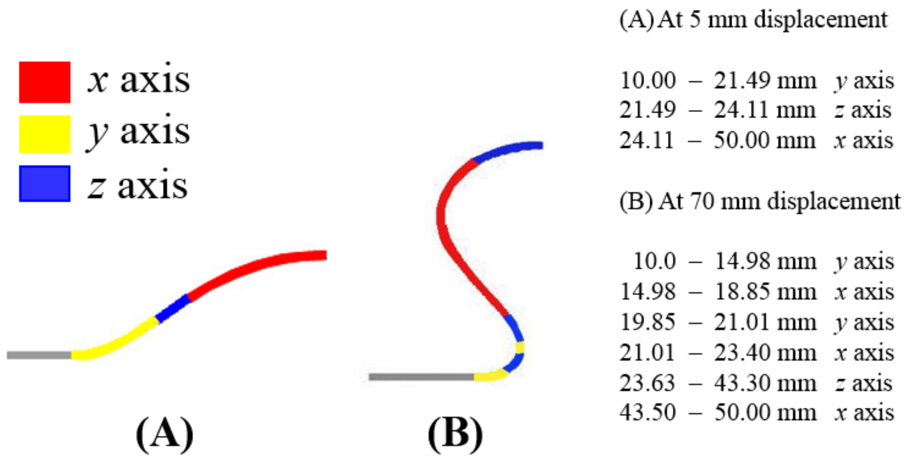

Figure S5. The direction of the Maximum Normal strain on the ribbon. We show the which direction (x,y,z) make the highest normal strain at each ribbon portion. (A) Result of 5 mm displacement (B) Result of 70 mm displacement. The direction of the maximum normal strain will be changed in different displacement. A red color shows the highest normal strain caused along x axis. (Yellow is y axis and blue is z axis) These results are used for determining the effective location to create the patterns.

Details of 2D ribbons

Table S1. Design details about 2D ribbons. (First row) Type of profiles; (Second row) Target displacement mm; (Third row) Number of ribbons; (Fourth row) Type of patterns; (Fifth row) Width of line partial-cuts; (Sixth row) Location of patterns (Seventh row); Thickness of ribbons from left to light; Strawberry and crown profiles are used pattern of linearly varying thickness.

**Name of profile**	**Target displacement (mm)**	**Ribbons**	**Pattern type**	**With of cut (mm)**	**Pattern location (mm)**	**Thickness Left to right (mm)**
Profile 1	16.5 mm	8	No	No	No	2 – 2
Profile 1.5	45.3 mm	8	No	No	No	2 – 2
Profile 0.5	4.8 mm	8	No	No	No	2 – 2
Kiwi	16.5 mm	8	Two line cuts	5 mm	25,75	2 – 2
Strawberry	16.5 mm	8	Thickness	Ratio2:1	Entire ribbon	2 – 1
Hourglass	8.3 mm	8	No	No	No	2 – 2
Spherical	16.5 mm	8	Two line cuts	5 mm	At 15, 85	2 – 2
Bi-cone	16.5 mm	8	One line cut	8 mm	At 50	3 – 3
Wheel	60 mm	16	Two line cuts	5 mm	At 30 70	2 – 2
Crown	60 mm	16	Thickness	Ratio2:1	Entire ribbon	3 – 1.5
Profile 16	16. 5mm	16	No	No	No	2 – 2
Egg shell	16.5 mm	16	Modified width	No	No	2 – 2

Patterns of linearly varying thickness

There is a limitation to create varieties of three-dimensional shape by using the ribbon, which does not contain any pattern on the surface since the profile change does not create a big difference in entire deformed shape. However, thickness change can greatly affect the deformed shape and is a very effective method for creating unique shape. The descending thickness change is changing the thickness linearly from one end of the ribbon to the other end. For example, the strawberry and crown profiles can be obtained with the patterns in figure 1 (Main text), and Figure S6 shows the deformed configurations of five different thickness ratios at three displacements. The curvature of the deformed shape increases as thickness ratio decreases since the thinner section causes relatively large curvature. Another finding is that when the thickness ratio is small, the deflection along the z axis reaches the maximum in early stage smaller displacement.

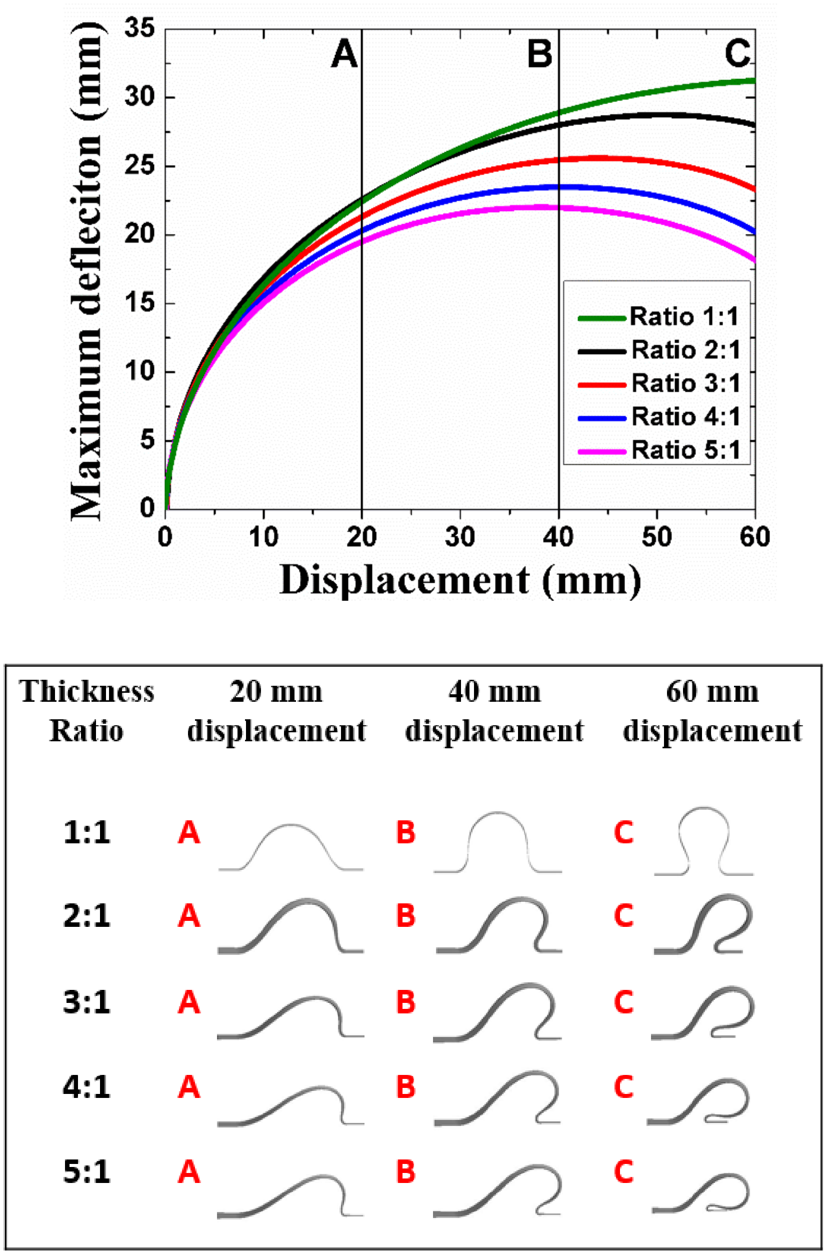

Figure S6. Deformed configuration in five-thickness ratios. (Top) Relationship between the maximum deflection and displacement (Effect of the thickness ratio). (Bottom) Deformed configurations at line A, B and C

Pattern of Line cut

The pattern of linearly varying thickness is applied for creating smooth asymmetrical three-dimensional shapes. (Asymmetrical about *y* axis) Another possible pattern is called line cut, which is a sudden thickness change in the ribbon. A line cut can only be applied to specific spots on a ribbon, and this pattern can create irregular bumps at deformed ribbons. In other words, a ribbon with line cuts applied to specific spots can trigger sudden curvature changes.

The profiles of kiwi and wheel are two examples where two line cuts are created on the ribbons as shown in Figure 1 (Main text). Five different line cut positions with three displacement levels contributes to the deformed configuration results shown in figure S7.The line cuts at 35 mm and 40 mm (the distance from the end of the ribbon) result in very different maximum deflections. Although the deformed shapes for the cases with line cut at 20 mm and 25 mm are fairly close, the cases with 30 mm and 35 mm are very different. At stage A, the differences are not obvious; however, the differences enlarge at stage B and C. We also observed that the change of maximum deflection does not correspond with the line cut position in order (25mm being the largest, 35mm the lowest, the rest falls in between).

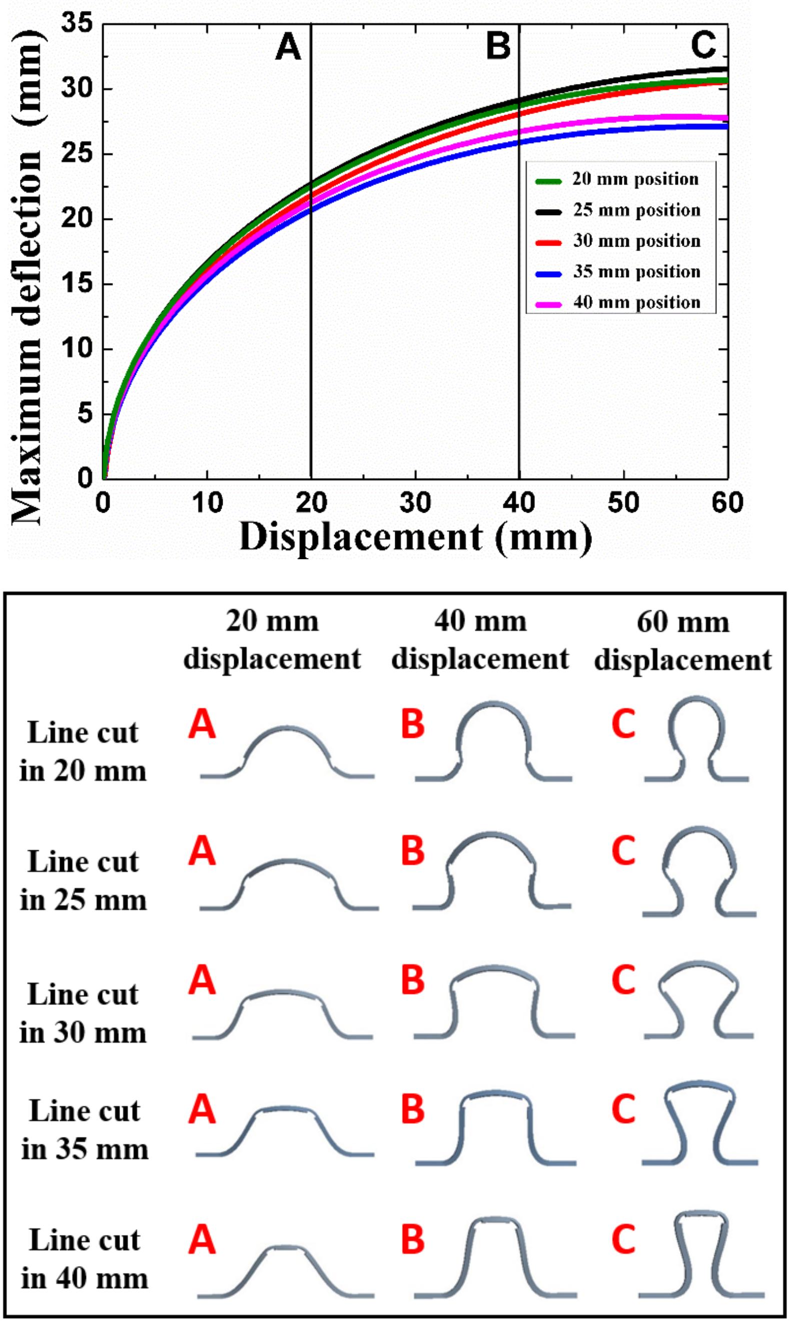

Figure S7. Deformed configurations in five positions of line cut (Top) Relationship between the maximum deflection and displacement (Effect of the line cut position) (Bottom) Deformed configurations at line A, B and C

The sequence of shape transformation from two-dimensional ribbon to three-dimensional.

Overlap portions are observed in reference configuration as shows in Figure S8 (A). In ANSYS WORKBENCH, the shape transformation of the two-dimensional ribbon can be achieved by applying the targeted displacement only since two different ribbons can penetrate each other in the computational software. However, this does not reflect the real transformational sequence. As a result, the special sequence must be considered to achieve the three-dimensional closed shape deformation. Each ribbon limits the shape deformation of the others. One possible method is applying the rotation on one fixed base to avoid overlapping. Following steps are shown to demonstrate the actual sequence from two-dimensional to three-dimensional.

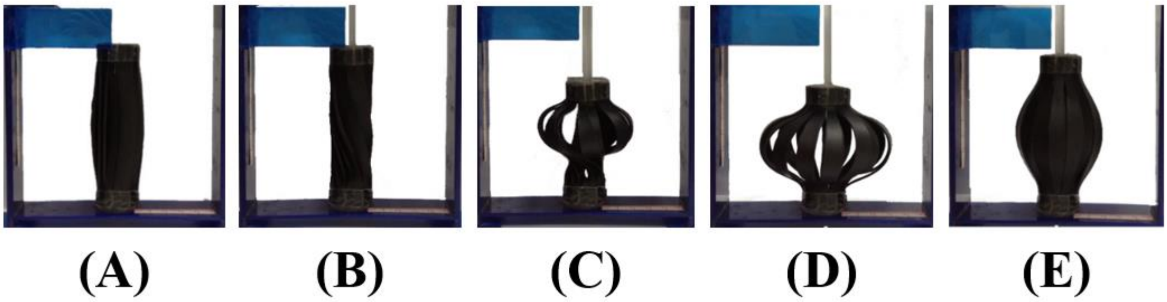

Figure S8. Experiment about sequence of shape transformation from two-dimensional to three-dimensional (A) The original configuration (B) Rotate in clockwise direction (C) Apply displacement (D) Rotate back to the original angle (E) Form to three-dimensional closed shape. Ribbons are used black thin sheets made of Thermoplastic Olefin (TPO).

Contact condition in different displacement

The figure introduces how much distance (gap and overlap) exists between the ideal contact positions and actual positions. (The figure S9 is result of the master profile 1.0×) The negative sign indicates the overlap and the positive sign indicates gap. The S10 shows the contact condition in various displacement.

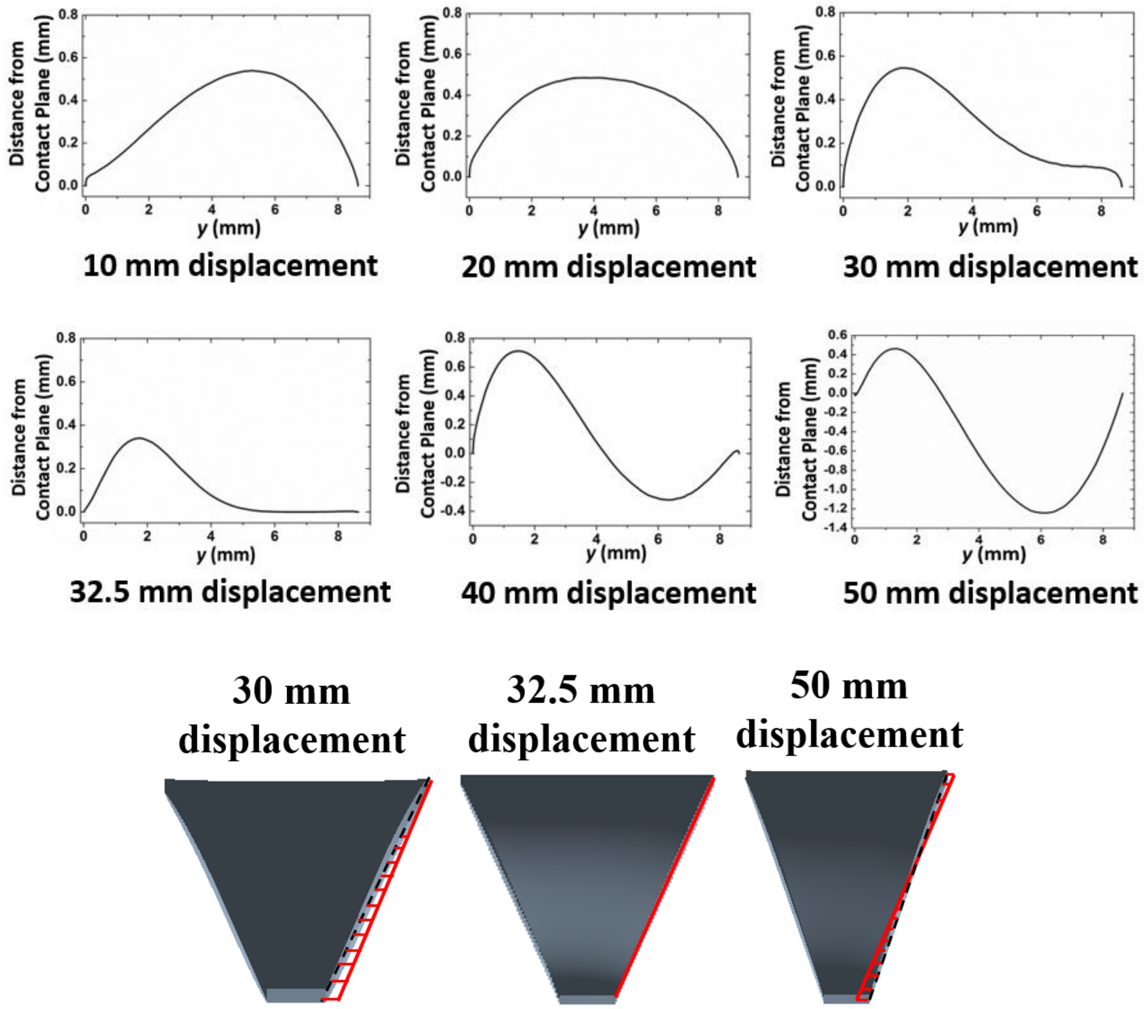

Figure S9. The contact condition in different displacement (Top) Displacement at six different spots (Bottom) Schematic diagram of contact condition in three displacement

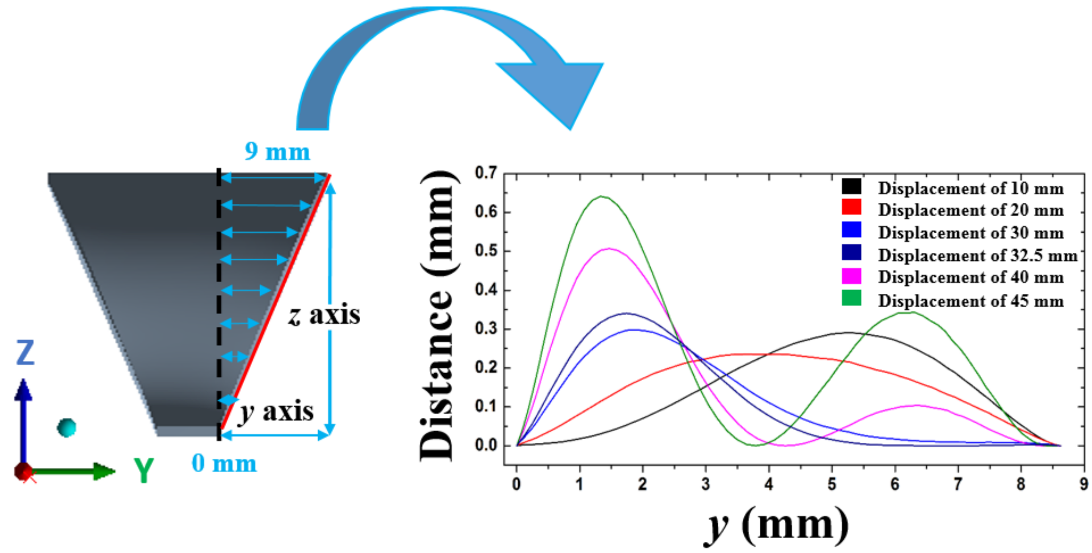

Figure S10. (Left) Schematic diagram of deformed ribbon (Right) Comparison of the contact condition in various displacement.

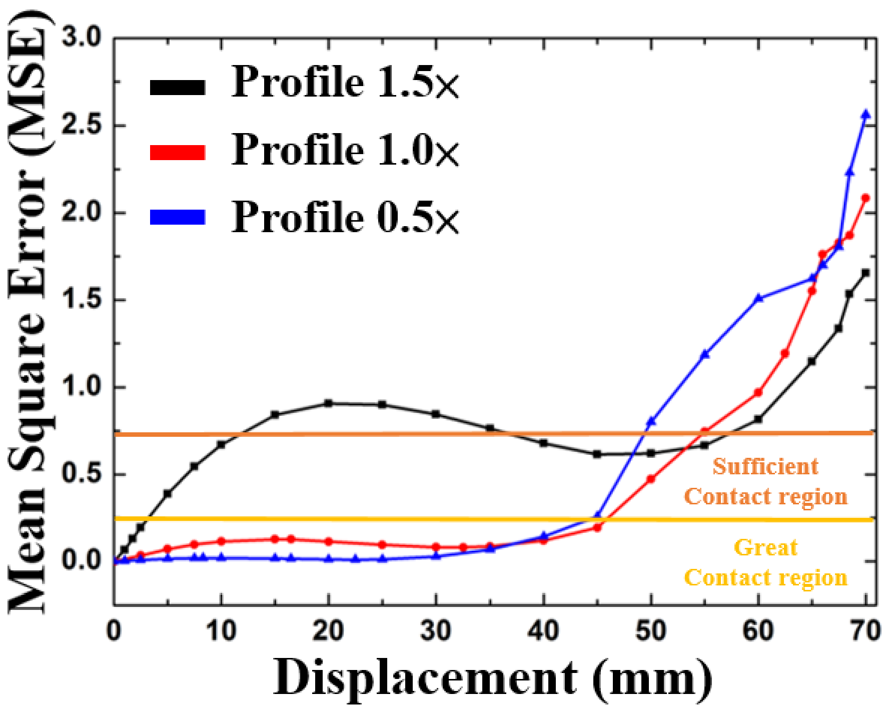

Figure S11. The Mean Squar Error (MSE) at the multiple displacemnt with profile 1.5×, 1.0× and 0.5×. The great contact region and sufficient contact region are illustrated.

Experimental devices

The device is used for measuring the size of displacement and maximum deflection in the single ribbon. The device is called “Extend table “that changes the rotational motion to liner motion. The stepping motor is set on the bottom of device, we make a simple algorithm to precisely control the size of displacement rather than manually move the fixing base.

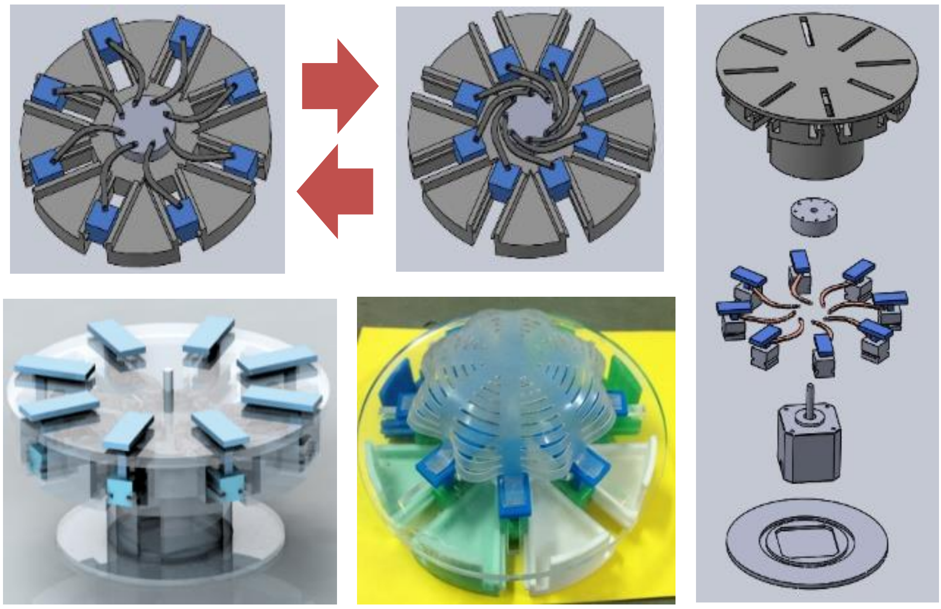

Figure S12. Experimental device for measuring the size of displacement. (Extend table)

The main purpose of this device is to make a 3D closed shape by using several identical ribbons. The experimental device needs to be designed with one side fixed to the bottom and the other stays adjustable to be moved toward the fixed side. The device is consisted of a main frame made with acrylic plates and two base parts in the center which are modifiable along the pole in the center. We use this device to make approximate comparison with the simulation results from the ANSYS Workbench.

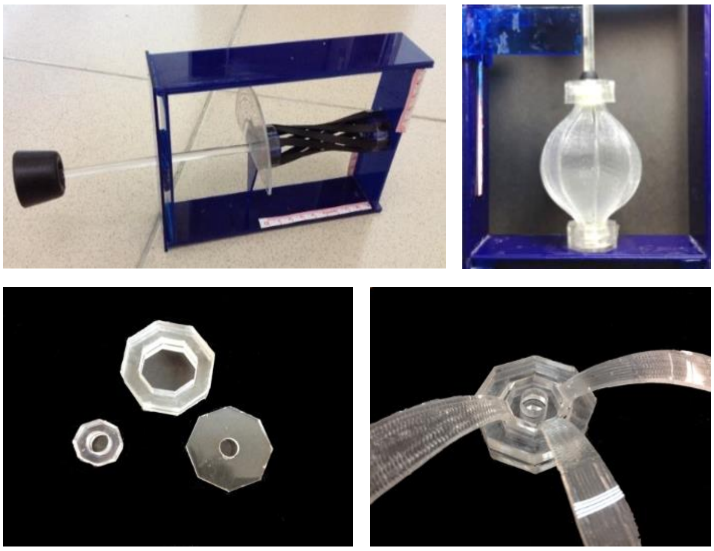

Figure S13. (Top) Experimental device of creating a fully closed shape (Bottom) Fixed base.

We extend the concept of fully closed shape to create application of LED lighting device. A frame work and basement are manufactured by digital design and fabrication tool of 3D printing. The soft light bulb is made of transparent elastomer and is illuminated when the light of an external source passes through its surface by total reflection. The figure below shows the prototype of the LED lighting device.

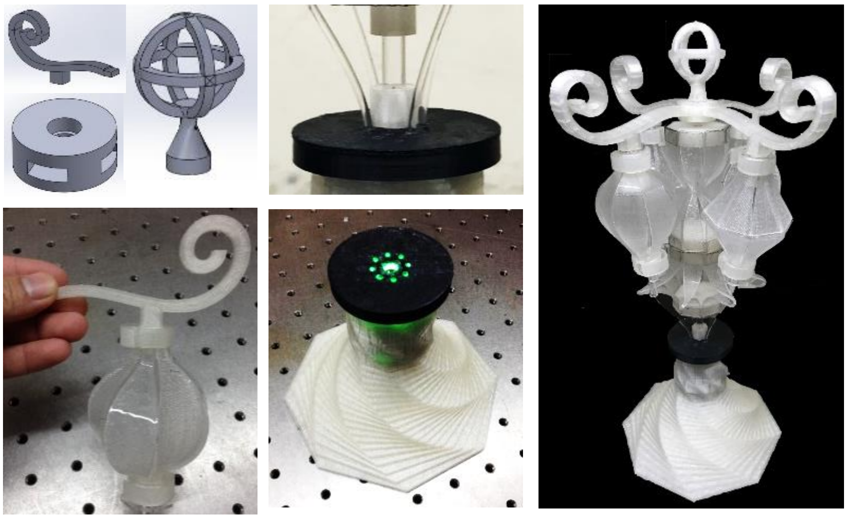

Figure S14. LED lamp with soft light bulbs

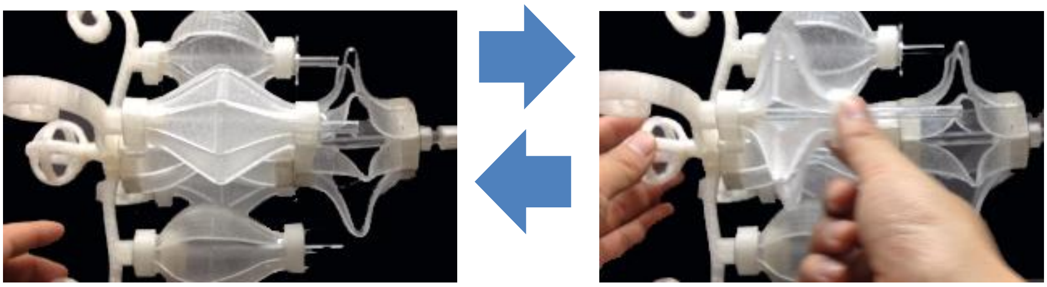

Figure S15. Shape transformation of the soft light bulb

Shape transformation of the soft light bulb

The same elastic ribbon design can be transformed into infinite 3D-shaped models simply by applying different displacements. Examples of three different 3D-shaped models obtained from an identical ribbon design. The models from the left to the right are applied with a displacement of 20 mm, 8 mm and no displacement, respectively**.**

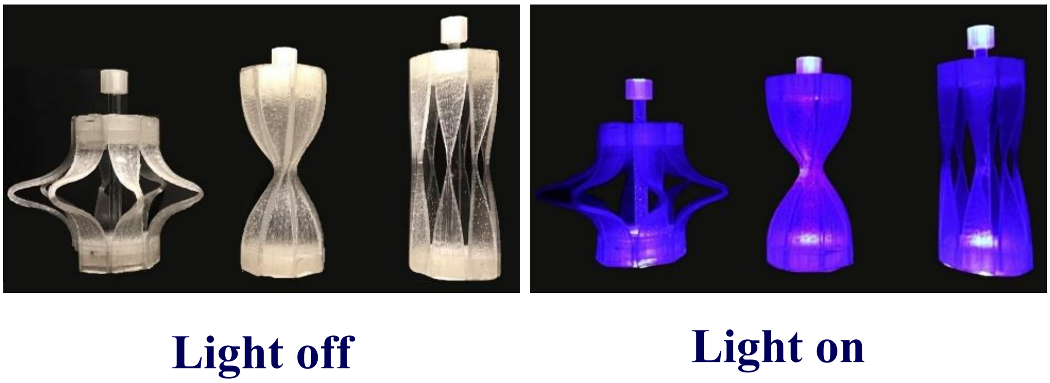

Figure S16. The same elastic ribbon design into infinite 3D shaped models by applying different displacements.

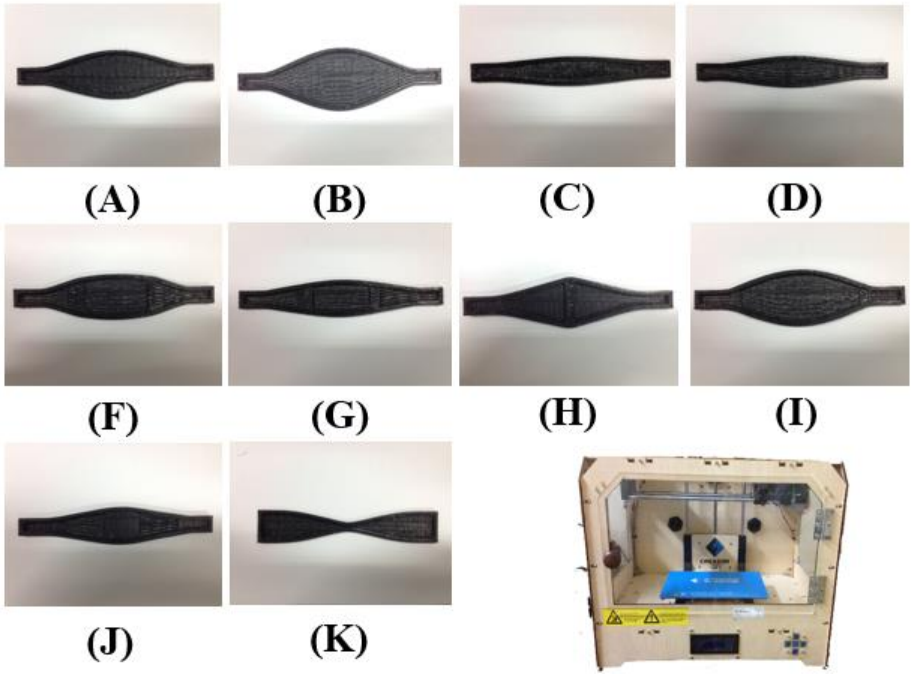

Figure S17. Mold design for PDMS (A) Profile size 1 (B) profile size 1.5 (C) profile size 0.5 (D) profile 16 ribbons (E) kiwi profile (F) Wheel profile (G) bi-cone profile (H) spherical profile (I) crown profile (J) Hourglass profile
